# Risankizumab, a therapeutic alternative for psoriasis in people living with HIV

**DOI:** 10.1177/03000605241229324

**Published:** 2024-03-12

**Authors:** Tomás Estevinho, Egídio Freitas, Tiago Torres

**Affiliations:** 1 Hospital do Divino Espírito Santo, Ponta Delgada, Portugal; 2 Department of Dermatology, Centro Hospitalar Universitário de Santo António, Porto, Portugal; 3 Instituto de Ciências Biomédicas Abel Salazar, 26706 University of Porto, Porto, Portugal

**Keywords:** Risankizumab, psoriasis, HIV, case report, biologic treatment, interleukin-23 inhibition

## Abstract

The management of psoriasis in individuals with human immunodeficiency virus (HIV) presents a unique challenge, marked by a more severe progression and limited efficacy of first- and second-line treatments. Although conventional systemic therapies might be considered, these agents are immunosuppressants, making their use challenging in people living with HIV (PLHIV). Biologic agents are frequently used in individuals with moderate-to-severe psoriasis, but their efficacy and safety data in PLHIV are very limited, as this patient group tends to be excluded from clinical trials. Risankizumab is a selective interleukin-23 (IL-23) inhibitor that has demonstrated a favourable safety profile and high efficacy in long-term studies and clinical practice. This current case report presents two clinical cases of PLHIV with plaque psoriasis who were effectively treated with the biologic agent risankizumab, with no reported safety issues. Although there are limited data on the use of biologics in PLHIV, this case series suggests that IL-23 inhibitors, namely risankizumab, might be a valuable therapeutic option for this population. Additional research and larger studies are needed to gain a more comprehensive understanding of the long-term safety and efficacy of IL-23 inhibitors in individuals affected by HIV.

## Introduction

While the prevalence of psoriasis in people living with human immunodeficiency virus (HIV) is similar to that of the general population, its progression tends to be more severe in individuals with HIV.^1,2^ In fact, HIV infection can be both a trigger and an exacerbating factor for psoriasis.^3,4^

Managing psoriasis in people living with HIV (PLHIV) presents a unique challenge. Topical therapies, highly active antiretroviral therapy (HAART) and phototherapy are the first-line therapeutic options in these individuals, with oral retinoids used as second-line treatment.^5^ However, in this patient group, the disease course is frequently marked by its severity and treatment resistance; therefore, these therapeutic approaches often prove insufficient in effectively controlling the clinical manifestations of psoriasis.^6^

Thus, other systemic therapies, namely conventional systemic agents (methotrexate and cyclosporine) and biologics, might be considered.^5^ Methotrexate and cyclosporine are associated with various adverse effects, including end-organ toxicity and drug–drug interactions, requiring close laboratory monitoring.^7^ Furthermore, these agents are immunosuppressants, making their use challenging in PLHIV, particularly those not receiving HAART, who already have a compromised immune status.^5,6^ Biologic agents are commonly used in patients with moderate-to-severe psoriasis, with excellent long-term and short-term efficacy and a favourable safety profile compared to conventional systemic therapies.^8,9^ However, the efficacy and safety data for biologics in PLHIV are very limited, as this patient group tends to be excluded from clinical trials.^10,11^

This current report presents a case series of two HIV-positive individuals with psoriasis who were effectively treated with risankizumab, a selective interleukin-23 (IL-23) inhibitor, without encountering any safety issues.

## Case reports

In March 2020, a 34-year-old woman (case 1) with severe plaque psoriasis and HIV infection with poor adherence to HAART presented to the Department of Dermatology, Centro Hospitalar Universitário de Santo António, Porto, Portugal. In addition, the patient was obese and had untreated dyslipidaemia. There was no family history of psoriasis and no personal history of psoriatic arthritis. She presented due to the worsening of her psoriasis, with a body surface area (BSA) of approximately 20% and a psoriasis area and severity index (PASI) of 24, along with an inadequate response to topical therapy. Her HIV viral load was 1230 copies/ml and her CD4 count was 12 cells/μl. Considering the patient's clinical context, the recommended dose of risankizumab (150 mg administered as a subcutaneous injection at week 0, week 4 and every 12 weeks thereafter) was initiated. After 8 weeks of treatment, she achieved a PASI 100 response; and no significant adverse events were reported during a 168-week follow-up. After initiating risankizumab and experiencing subsequent improvement in her skin condition, she started HAART (800/150/200/10 mg of darunavir/cobicistat/emtricitabine/tenofovir plus 50 mg of dolutegravir taken orally once daily) with good adherence, resulting in an undetectable viral load and an increase in her CD4 count to over 300 cells/μl.

**Figure 1.**
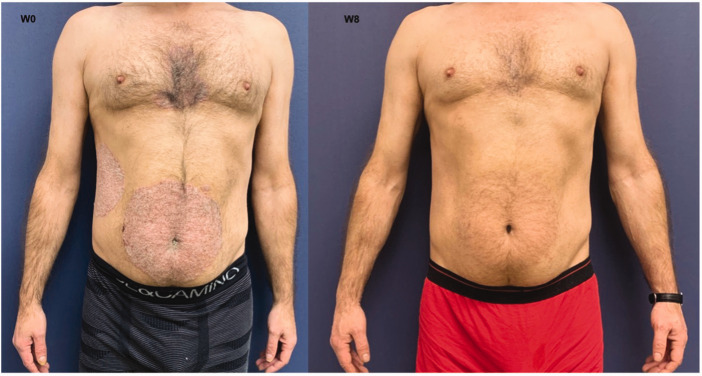
Photographs of a 35-year-old male patient (case 2) who presented with worsening plaque psoriasis and a concomitant HIV infection managed with highly active antiretroviral therapy showing the psoriatic plaques before starting treatment with risankizumab (W0) and after 8 weeks of therapy (W8). The colour version of this figure is available at: http://imr.sagepub.com.

In October 2022, a 35-year-old man (case 2) with a long-standing history of plaque psoriasis and HIV infection managed with HAART (50/300 mg of dolutegravir/lamivudine taken orally once daily) presented to the Department of Dermatology, Centro Hospitalar Universitário de Santo António, Porto, Portugal. There was no personal history of other comorbidities and no family history of psoriasis. He presented due to the worsening of his psoriasis, with a BSA of approximately 22% and a PASI of 21, along with an inadequate response to topical therapies and 25 mg acitretin taken orally once daily. His HIV viral load was undetectable and his CD4 count was 512 cells/μl. Starting with the recommend risankizumab dose (150 mg administered as a subcutaneous injection at week 0, week 4 and every 12 weeks thereafter), a PASI 100 response was achieved at week 8 (Figure 1); and no significant adverse events were reported during a 56-week follow-up. He maintained an undetectable viral load and a CD4 count within the same range as pre-treatment.

Both patients provided written informed consent for both their treatment and the publication of this case report. It should be noted that the treatment decisions were made in cooperation with an HIV specialist. The reporting of these two cases conforms to the CARE guidelines.^12^

## Discussion

Interleukin-23 plays a major role in the pathogenesis of psoriasis by promoting the differentiation and proliferation of T helper 17 cells, which, upon activation, produce several pro-inflammatory cytokines such as IL-17, the most important effector cytokine in psoriasis.^13,14^ Risankizumab binds with high affinity to IL-23 via its unique p19 subunit, effectively inhibiting the inflammatory cascade triggered by this key cytokine.^15^

Due to their immunosuppressive potential, the long-term use of biologics in immunocompromised individuals (such as PLHIV) raises some safety concerns, including an increased risk of infections.^16,17^ Although data remain limited, regarding the use of biologics in the HIV population, existing evidence supports the therapeutic efficacy and safety of these agents, namely IL-23, IL-17 and tumour necrosis factor (TNF) inhibitors, for treating HIV-positive individuals with moderate-to-severe psoriasis.^18–20^ Furthermore, evidence suggests that selective inhibition of IL-23, particularly through its unique p19 subunit, provides a more favourable safety profile when compared with inhibiting other inflammatory cytokines such as IL-17 and TNF.^17^ In fact, a recent meta-analysis concluded that risankizumab has the most favourable long-term benefit–risk profile compared with other biologic agents for the treatment of psoriasis.^21^

Seven cases of PLHIV with psoriasis treated with risankizumab have been reported (Table 1).^22–24^ In all seven cases, the treatment with risankizumab was effective, well-tolerated and no significant adverse events were recorded.^22–24^ In addition, a PLHIV with psoriasis treated with guselkumab, another IL-23 inhibitor, has been reported (Table 1).^25^ The treatment proved effective, with no reported adverse events, and both viral load and CD4 count remained stable throughout the treatment period.^25^

**Table 1.** Case reports of people living with human immunodeficiency virus (HIV) and concomitant psoriasis treated with interleukin-23 inhibitors.

Study	Sex/Age	Therapy	HAART	CD4 count, cells/μl		HIV viral load, copies/ml		Significant AE	PASI	
				Pre-treatment	Post-treatment	Pre-treatment	Post-treatment		Pre-treatment	Post-treatment
Rob & Rozsypal 2022^22^	M/36	Risankizumab	Yes	N/S	630	Undetectable	Undetectable	None	10.3	0 (after 12 weeks)
Maliyar et al. 2023^23^	M/36	Risankizumab	Yes	1461	1225	Undetectable	Undetectable	None	5.4	0 (after 5 months)
	M/58	Risankizumab	Yes	831	926	Undetectable	Undetectable	None	4.0	1.6 (after 3 doses)
Orsini et al. 2023^24^	M/53	Risankizumab	Yes	N/S	N/S	N/S	NVR	None	22	0 (after 16 weeks)
	M/32	Risankizumab	Yes	N/S	N/S	N/S	NVR	None	30	0 (after 16 weeks)
	M/58	Risankizumab	Yes	439	N/S	Undetectable	NVR	None	20	1 (after 16 weeks)
	M/53	Risankizumab	Yes	N/S	N/S	Undetectable	NVR	None	10	0 (after 16 weeks)
Bartos et al. 2018^25^	M/51	Guselkumab	Yes	54	41	29	Undetectable	None	N/S	0 (after 6 months)
This current case series	F/34	Risankizumab	No	12	> 300	1230	Undetectable	None	24	0 (after 8 weeks)
	M/35	Risankizumab	Yes	512	Stable	Undetectable	Undetectable	None	21	0 (after 8 weeks)

HAART, highly active antiretroviral therapy; AE, adverse events; PASI, psoriasis area and severity index; M, male; N/S, not specified; NVR, no viral reactivation; F, female.

Despite limited data on the use of biologics in PLHIV, this case series suggests that IL-23 inhibitors, such as risankizumab, might be a valuable therapeutic choice for this particular population. The positive balance between benefits and risks, observed in both long-term studies and case reports of PLHIV (Table 1), reinforces their potential as effective and safe treatments for psoriasis in individuals with HIV.^22–25^

Further research and larger studies involving this specific cohort are needed to better understand the long-term safety and efficacy of IL-23 inhibitors in people affected by HIV. Nevertheless, these findings contribute to the progression and evolution of psoriasis management in immunocompromised individuals, providing hope for better outcomes in this challenging patient group.

In conclusion, managing psoriasis in PLHIV poses a unique challenge due to the frequently severe course of the disease and the limitations of conventional therapies in this patient group. These two current patients, one with poor adherence to HAART and the other effectively managed with HAART, experienced significant improvement of their psoriasis with risankizumab therapy, with no significant adverse events reported during follow-ups of 168 and 56 weeks, respectively.

## References

[1] Queiros N Torres T. HIV-Associated Psoriasis. Actas Dermosifiliogr (Engl Ed) 2018; 109: 303–311. DOI: 10.1016/j.ad.2017.09.014. 29361272 10.1016/j.ad.2017.09.014

[2] Mamkin I Mamkin A Ramanan SV. HIV-associated psoriasis. Lancet Infect Dis 2007; 7: 496. DOI: 10.1016/S1473-3099(07)70161-5. 17597572 10.1016/S1473-3099(07)70161-5

[3] Arbune M Arbune AA Niculet E , et al. Therapeutic challenges of psoriasis in the HIV-infected patient: A case report. Exp Ther Med 2022; 23: 175. DOI: 10.3892/etm.2021.11098. 35069856 10.3892/etm.2021.11098 PMC8764576

[4] Ceccarelli M Venanzi Rullo E Vaccaro M , et al. HIV-associated psoriasis: Epidemiology, pathogenesis, and management. Dermatol Ther 2019; 32: e12806. DOI: 10.1111/dth.12806. 30588732 10.1111/dth.12806

[5] Menon K Van Voorhees AS Bebo BF Jr , et al. Psoriasis in patients with HIV infection: from the medical board of the National Psoriasis Foundation. J Am Acad Dermatol 2010; 62: 291–299. DOI: 10.1016/j.jaad.2009.03.047. 19646777 10.1016/j.jaad.2009.03.047

[6] Nakamura M Abrouk M Farahnik B , et al. Psoriasis treatment in HIV-positive patients: a systematic review of systemic immunosuppressive therapies. Cutis 2018; 101: 38–42. 56. PMID: 29529104. 29529104

[7] Balak DMW Gerdes S Parodi A , et al. Long-term Safety of Oral Systemic Therapies for Psoriasis: A Comprehensive Review of the Literature. Dermatol Ther (Heidelb) 2020; 10: 589–613. DOI: 10.1007/s13555-020-00409-4. 32529393 10.1007/s13555-020-00409-4 PMC7367959

[8] Torres T Velho GC Sanches M , et al. Psoriasis in the era of biologics. Acta Med Port 2010; 23: 493–498 [Article in Portuguese, English abstract]. PMID: 20654269. 20654269

[9] Zweegers J Otero ME van den Reek JM , et al. Effectiveness of Biologic and Conventional Systemic Therapies in Adults with Chronic Plaque Psoriasis in Daily Practice: A Systematic Review. Acta Derm Venereol 2016; 96: 453–458. DOI: 10.2340/00015555-2276. 26537336 10.2340/00015555-2276

[10] Xu J Gill K Flora A , et al. The impact of psoriasis biologic therapy on HIV viral load and CD4(+) cell counts in HIV-positive individuals: A real-world cohort study. J Eur Acad Dermatol Venereol 2023; 37: 1659–1663. DOI: 10.1111/jdv.19020. 10.1111/jdv.19020 36897246

[11] Plachouri KM Georgiou S. Challenges in the treatment of psoriasis with biologics: vaccination, history of malignancy, human immunodeficiency virus (HIV) infection, and pediatric psoriasis. Int J Dermatol 2019; 58: 1008–1013. DOI: 10.1111/ijd.14436. 30891751 10.1111/ijd.14436

[12] Gagnier JJ Kienle G Altman DG , et al. The CARE Guidelines: Consensus-based Clinical Case Reporting Guideline Development. Glob Adv Health Med 2013; 2: 38–43. DOI: 10.7453/gahmj.2013.008. 10.7453/gahmj.2013.008 PMC3833570 24416692

[13] Alwan W Nestle FO. Pathogenesis and treatment of psoriasis: exploiting pathophysiological pathways for precision medicine. Clin Exp Rheumatol 2015; 33: S2–S6. PMID: 26472336. 26472336

[14] Hawkes JE Yan BY Chan TC , et al. Discovery of the IL-23/IL-17 Signaling Pathway and the Treatment of Psoriasis. J Immunol 2018; 201: 1605–1613. DOI: 10.4049/jimmunol.1800013. 30181299 10.4049/jimmunol.1800013 PMC6129988

[15] Torres T. Selective Interleukin-23 p19 Inhibition: Another Game Changer in Psoriasis? Focus on Risankizumab. Drugs 2017; 77: 1493–1503. DOI: 10.1007/s40265-017-0794-1. 28770513 10.1007/s40265-017-0794-1

[16] Kalb RE Fiorentino DF Lebwohl MG , et al. Risk of Serious Infection With Biologic and Systemic Treatment of Psoriasis: Results From the Psoriasis Longitudinal Assessment and Registry (PSOLAR). JAMA Dermatol 2015; 151: 961–969. DOI: 10.1001/jamadermatol.2015.0718. 25970800 10.1001/jamadermatol.2015.0718

[17] Blauvelt A Chiricozzi A Ehst BD , et al. Safety of IL-23 p19 Inhibitors for the Treatment of Patients With Moderate-to-Severe Plaque Psoriasis: A Narrative Review. Adv Ther 2023; 40: 3410–3433. DOI: 10.1007/s12325-023-02568-0. 37330926 10.1007/s12325-023-02568-0 PMC10329957

[18] Myers B Thibodeaux Q Reddy V , et al. Biologic Treatment of 4 HIV-Positive Patients: A Case Series and Literature Review. J Psoriasis Psoriatic Arthritis 2021; 6: 19–26. DOI: 10.1177/2475530320954279. 35784673 PMC9249044

[19] Pangilinan MCG Sermswan P Asawanonda P. Use of Anti-IL-17 Monoclonal Antibodies in HIV Patients with Erythrodermic Psoriasis. Case Rep Dermatol 2020; 12: 132–137. DOI: 10.1159/000508781. 32999648 10.1159/000508781 PMC7506271

[20] Gong J Wu W Qiu L , et al. Interleukin-17A Inhibitor Secukinumab Treatment in HIV-Positive Psoriasis Patient: A Case Report. Clin Cosmet Investig Dermatol 2022; 15: 2949–2956. DOI: 10.2147/CCID.S395348. 10.2147/CCID.S395348 PMC9809383 36605452

[21] Armstrong AW Soliman AM Betts KA , et al. Long-Term Benefit-Risk Profiles of Treatments for Moderate-to-Severe Plaque Psoriasis: A Network Meta-Analysis. Dermatol Ther (Heidelb) 2022; 12: 167–184. DOI: 10.1007/s13555-021-00647-0. 34862951 10.1007/s13555-021-00647-0 PMC8776931

[22] Rob F Rozsypal H. Successful treatment of psoriasis with risankizumab in an HIV positive patient with sexually transmitted infection comorbidities. Dermatol Ther 2022; 35: e15277. DOI: 10.1111/dth.15277. 34923721 10.1111/dth.15277

[23] Maliyar K Lansang P Doiron P. Use of risankizumab in two HIV-positive patients with psoriasis. JAAD Case Rep 2023; 33: 54–55. DOI: 10.1016/j.jdcr.2023.01.015. 36860809 10.1016/j.jdcr.2023.01.015 PMC9969266

[24] Orsini D Maramao FS Gargiulo L , et al. Effectiveness and safety of risankizumab in HIV patients with psoriasis: A case series. Int J STD AIDS 2024; 35: 67–70. DOI: 10.1177/09564624231199510. 37691387 PMC10751968

[25] Bartos G Cline A Beroukhim K , et al. Current biological therapies for use in HIV-positive patients with psoriasis: case report of gesulkumab used and review. Dermatol Online J 2018; 24: 10.5070/D32411041999. 30695971

